# Data-driven nonlinear model reduction to spectral submanifolds in mechanical systems

**DOI:** 10.1098/rsta.2021.0194

**Published:** 2022-08-08

**Authors:** M. Cenedese, J. Axås, H. Yang, M. Eriten, G. Haller

**Affiliations:** ^1^ Institute for Mechanical Systems, ETH Zürich, Leonhardstrasse 21 8092, Zürich, Switzerland; ^2^ Department of Mechanical Engineering, University of Wisconsin-Madison, 1513 University Avenue, Madison, WI 53706, USA

**Keywords:** nonlinear dynamics, mechanical vibrations, reduced-order modelling, normal form, machine learning

## Abstract

While data-driven model reduction techniques are well-established for linearizable mechanical systems, general approaches to reducing nonlinearizable systems with multiple coexisting steady states have been unavailable. In this paper, we review such a data-driven nonlinear model reduction methodology based on spectral submanifolds. As input, this approach takes observations of unforced nonlinear oscillations to construct normal forms of the dynamics reduced to very low-dimensional invariant manifolds. These normal forms capture amplitude-dependent properties and are accurate enough to provide predictions for nonlinearizable system response under the additions of external forcing. We illustrate these results on examples from structural vibrations, featuring both synthetic and experimental data.

This article is part of the theme issue ‘Data-driven prediction in dynamical systems’.

## 1. Introduction

Dimensionality reduction for datasets representing high-dimensional nonlinear mechanical systems is of crucial importance in science and technology. Low-dimensional models are expected to reduce computational cost and capture the essential physics of a high-dimensional system from data. Given the growing interest, for example, in light mechanical structures and MEMS devices, there is the need for truly nonlinear models, capturing amplitude-dependent properties and competing steady-states solutions, which are increasingly important to identify, as highlighted in experiments of nonlinear mechanical vibrations [1–8]. Predicting coexisting stable and unstable forced responses for a broad range of forcing amplitudes and frequencies is paramount in structural dynamics. However, a generally applicable technique returning such reliable low-dimensional models of nonlinear mechanical vibrations has not emerged yet.

The most common approaches to data-driven reduced-order modelling are the proper orthogonal decomposition (POD) followed by a Galerkin projection [9–13] and the dynamic mode decomposition (DMD) [14,15]. The former approach requires the knowledge of the governing equations of motion and, once a relevant number of modes is identified from data, projects these equations onto those modes to construct a reduced-order model. DMD and its improved versions [16–22], supported by Koopman operator theory [23,24], seek a low-rank approximation to the dynamics of observable data without reliance on the governing equations of motion. With this approach, DMD and Koopman mode expansions are able to linearize the observed dynamics around attracting fixed points on domains that cannot include additional fixed points or limit cycles [25–28]. Therefore, while truly powerful for globally linearizable dynamics [29], these linear techniques cannot capture essentially nonlinear dynamical systems (or *nonlinearizable* systems) with multiple coexisting steady states.

Other approaches treat the dimensionality reduction and the data-driven dynamical modelling as separate problems. Typically, the data are first processed via dimensionality reduction algorithm, which ranges from POD or principal component analysis (PCA) [30], its kernelized version [31], subspace adaptation [32], manifold learning techniques [33–35] or autoencoders [36,37]. Structural dynamics problems admit very often a low-rank representation as only some modes are present in the system response [38]. Afterwards, the dynamics are identified in the reduced coordinates using classic regression techniques (least-squares [31], LASSO [39], SINDy [40]), Bayesian learning techniques [41] or neural networks in different architectures (fully connected, convolutional, recurrent) [36,42–44]. Some of these techniques return complex, black-box models (which may be non-physical [45]), while others offer sparse models (LASSO, SINDy, Bayesian learning), which allow for easy interpretation and analysis [40]. The resulting dynamics, however, are intrinsically determined by the representation offered by dimensionality reduction algorithms, unless penalized in the optimization [37]. Indeed, the advocated simplicity of sparse models depends critically on the reduction method, as even a linear coordinate change will dramatically destroy the sparsity of a model. In addition, those methods feature a high number of hyperparameters that need to be tuned extensively for good performance. Most importantly, the eventual lack of predictive capabilities often makes the models unattractive for practical use. Indeed, the insertion of parameter variations, disturbances or external forcing into these models is generally heuristic, and hence returns questionable conclusions.

Recent machine-learning methods have increasingly been influenced by physics to address issues with interpretability and prediction [45,46]. The proposed tools include sparse regression [47,48], neural networks [49], neural ordinary differential equations [50], simultaneous basis function approximation and parameter estimation [51] (see [45] for an extensive review). These physics-informed models are easier to generalize and also handle sparse and incomplete data better.

Our objective here is to discuss a new data-driven reduced-order modelling approach in the context of mechanical vibrations, which is dynamics-based rather than physics-informed. Built on the recent theory of spectral submanifolds (SSMs) [52], this approach identifies very low dimensional, sparse models over different time scales by restricting the full system dynamics to a nested family of attractors. The SSMs forming this family are the smoothest nonlinear continuations of the eigenspaces of the linear part of the dynamical system. When transformed to a normal form, the reduced dynamics on each SSM is low-dimensional, sparse and relevant for all trajectories in the domain of attraction of the SSM. Importantly, each SSM may contain multiple coexisting steady states and hence capture nonlinearizable dynamics.

The details and several applications of SSM theory are discussed in [52–60] and an open-source Matlab^®^ implementation, SSMTool, for an arbitrary, finite-dimensional dynamical system is available in [61]. Another concept closely linked to SSMs is that of invariant foliations [62], which provides a rigorous nonlinear extension of classic linear modal analysis. Our present discussion of data-driven SSM-based models follows the terminology and notation of the more technical exposition in [63]. In contrast to [63], we focus here specifically on SSMs in mechanical systems and also give the first data-driven construction of higher-dimensional SSMs both with and without resonances.

The remainder of this paper is organized as follows. Section 2 introduces SSMs and discusses their relevance for data-driven model reduction, also depending on the type of experiments that generate the data. We also discuss how our method is complementary to (non-parametric) signal processing techniques in nonlinear system identification [64], ranging from the Hilbert transform and its variants [65,66] to wavelet decompositions [67]. In §2, we summarize our data-driven identification of SSMs and the resulting explicit models on SSMs. We demonstrate the method in §3 on a set of examples, which are all analysed via the Matlab^®^ implementation of our approach SSMLearn. In particular, after a preliminary numerical example, we examine two experimental datasets of nonlinear mechanical systems, one of which regards an internally resonant structure. The datasets in these examples come from diverse sources, from non-contact measurement systems (e.g. digital image correlation (DIC), laser scanner vibrometry) to classic accelerometers.

## 2. Spectral submanifolds and data-driven models on them

In this paper, we consider N-degree-of-freedom mechanical systems of the form

2.1 $$\text{M}(\text{q})\ddot{\text{q}}=\text{f}(\text{q},\dot{\text{q}}),\;\text{f}(\text{0},\text{0})=\text{0},\;\text{q}\in R^{N},\;N\geq 1,$$

where q is a generalized coordinate vector, $\text{M}(\text{q})\in R^{N\times N}$ is a positive definite, symmetric mass matrix. The forcing vector $\text{f}(\text{q},\dot{\text{q}})$ contains all conservative and non-conservative forces, including linear and nonlinear ones. The matrix M(q), its inverse and $\text{f}(\text{q},\dot{\text{q}})$ are of class $C^{r}$ with $r\in N^{+}\cup \{\infty \}$ (smooth functions) or r=a (analytic functions).

The equivalent first-order form of equation (2.1), with $\text{x}=(\text{q},\dot{\text{q}})\in R^{n}$ and n=2N, reads

2.2 $$\dot{\text{x}}=\text{A}\text{x}+\left(\begin{array}{c} \text{0}\\ \text{b}(\text{x}) \end{array}\right),\;\text{A}=\left[\begin{array}{cc} \text{0} & \text{I}\\ {\text{M}}^{-1}(\text{0})D_{\text{q}}\text{f}(\text{0},\text{0}) & {\text{M}}^{-1}(\text{0})D_{\dot{\text{q}}}\text{f}(\text{0},\text{0}) \end{array}\right],$$

where $\text{b}(\text{x})={\text{M}}^{-1}(\text{q})\text{f}(\text{q},\dot{\text{q}})-{\text{M}}^{-1}(\text{0})D_{\text{q}}\text{f}(\text{0},\text{0})\text{q}-{\text{M}}^{-1}(\text{0})D_{\dot{\text{q}}}\text{f}(\text{0},\text{0})\dot{\text{q}}$. We assume that x=0 is an asymptotically stable equilibrium and that A is a semi-simple matrix and has N complex conjugate pairs of eigenvalues with negative real parts. We order these eigenvalues $\lambda_{1},{\bar{\lambda }}_{1},\lambda_{2},{\bar{\lambda }}_{2},\ldots ,\lambda_{N},{\bar{\lambda }}_{N}$ with decreasing real parts, and we denote by $E_{1},E_{2},\ldots ,E_{N}$ the corresponding two-dimensional eigenspaces (or modal subspaces).

We denote by $E^{2m}$ the direct sum ⊕ of m of these modal subspaces, i.e. $E^{2m}=E_{j_{1}}⊕E_{j_{2}}⊕\cdots ,⊕E_{j_{m}}$. The 2m-dimensional, spectral subspace $E^{2m}$ is invariant for the linearization of system (2.2). Its reduced dynamics is governed by the eigenvalues $\lambda_{j_{1}},\lambda_{j_{2}},\ldots ,\lambda_{j_{m}}$, which, along with the conjugate ones, form the set $\mathrm{Spect}(\text{A}{|}_{E^{2m}})$. The SSM, $W(E^{2m})$, is the smoothest nonlinear continuation of the linear subspace $E^{2m}$ [52], as can be deduced from the more abstract invariant manifold results of [68–71]. Specifically, $W(E^{2m})$ is the unique 2m-dimensional, class $C^{r}$ invariant manifold of system (2.2) tangent to the spectral subspace $E^{2m}$ at the origin. The existence of $W(E^{2m})$ is guaranteed whenever the eigenvalues $\left(\lambda_{j},{\bar{\lambda }}_{j}\right)$ with $j\neq j_{1},j_{2},\ldots ,j_{m}$ are not in resonance with those in $\mathrm{Spect}(\text{A}{|}_{E^{2m}})$, i.e. for $\text{k}=(k_{1},k_{2},\ldots ,k_{2m})\in N^{2m}$,

2.3 λj−∑l=1m(λjlkl+λ¯jlkl+m)≠0,∑l=12mkl≤Int[minλ∈Spect(A)−Spect(A|E2m)Re(λ)maxλ∈Spect(A|E2m)Re(λ)],

as discussed in [52]. From a numerical perspective, the non-resonance condition in equation (2.3) is violated if the absolute value of the left-hand side of the inequality is below a certain tolerance. In that case, one needs to add the resonant modal subspace $E_{j}$ to $E^{2m}$, resulting in the SSM of the form $W(E^{2m}⊕E_{j})$. This larger SSM can be used to capture nonlinear modal interactions, e.g. in weakly damped systems with rationally dependent linearized frequencies. The dynamics restricted to SSMs gives exact nonlinear reduced-order models for system (2.2) [52,56,60].

**Figure 1.**
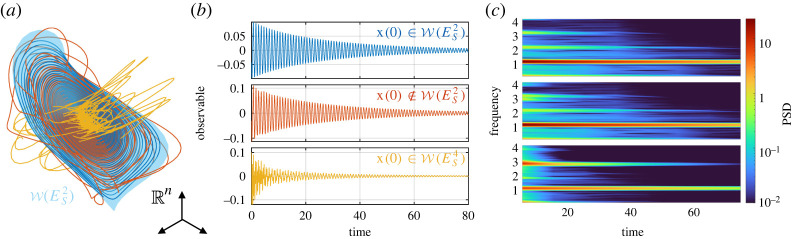
Illustration of different time scales of nonlinear dynamics captured by a nested set of SSMs. (*a*) Dynamics near a slow two-dimensional SSM $W(E_{S}^{2})$ with three trajectories in the phase space. (*b*,*c*) The three trajectories, shown with consistent colours, and their spectrogram. These trajectories were generated by a mechanical system in which 1,2.7,4.2 are the first three linearized frequencies. In (*b*,*c*), the top trajectory has initial condition x(0) on the slow two-dimensional SSM $W(E_{S}^{2})$ and decays on it, the middle is initialized with a small perturbation off $W(E_{S}^{2})$ and the bottom one decays on $W(E_{S}^{4})$. (Online version in colour.)

The most important SSMs from a data-driven perspective are slow SSMs, which are constructed over the spectral subspace spanned by the m slowest modes: $E_{S}^{2m}=E_{1}⊕E_{2}⊕\cdots ⊕E_{m}$. Slow SSMs are attracting normally hyperbolic invariant manifolds [52] to which nearby trajectories converge exponentially fast [72], as illustrated in figure 1*a*. Therefore, generic experiments on mechanical systems in the form of (2.2) will yield trajectories converging exponentially fast to slow SSMs, which in turn capture the asymptotic dynamics near the equilibrium. Faster timescales of the dynamics can be extracted from trajectory data by model reduction to higher-dimensional members of the nested slow SSM family $W(E_{S}^{2})\subset W(E_{S}^{4})\subset \cdots \subset W(E_{S}^{2(n-1)})\subset R^{n}$. As an illustration, trajectories on slow SSMs are shown in figure 1*b*, along with their spectrogram (or short time Fourier transform) in figure 1*c*. The trajectory on the top is in $W(E_{S}^{2})$, while the middle one is initialized close to $W(E_{S}^{2})$ and hence converges to $W(E_{S}^{2})$, as seen from the disappearance of higher frequencies. Finally, the trajectory at the bottom in figure 1*b*,*c* belongs to $W(E_{S}^{4})$, where two modal contributions can be clearly identified.

For trajectories with generic initial conditions—such as those generated by hammer impacts—discarding the initial part of the measured signal yields trajectory data close to a slow 2m-dimensional SSM, where m is the number of dominant frequencies in the signal. This number m is inferred from a preliminary time-frequency analysis (e.g. spectrograms, wavelet transforms [67]), such as those in figure 1*c*. The larger m, the more data is needed to properly explore the SSM, since for a well-posed model training data should contain enough nonlinear content for each mode and for eventual modal interactions. In principle, lower dimensions can be chosen if modelling the fastest transients is not of interest. By contrast, targeted experiments can focus on specific SSMs, which is the case for two-dimensional SSMs in resonance decay experiments [1,4]. In that setting, a near-resonant oscillation is first isolated using a shaker, which is then turned off. This generates a system trajectory that decays towards the equilibrium along the targeted two-dimensional SSM, provided that no internal resonance occurs. Using the shaker, we can typically excite higher amplitudes in comparison to hammer impact testing, where the energy is spread among multiple modes.

### (a) Learning spectral submanifolds from data

To learn SSMs from data, we use the methodology presented in [63], which is implemented in the open-source Matlab^®^ package, SSMLearn. In what follows, we sketch the main ideas of this method before going into the details of the data-driven reduced-order models that SSMLearn can identify.

Measuring all phase space variables of a mechanical system is generally unrealistic. Typically, only a limited set of observed quantities is available, so that we need to embed the SSM, $W(E^{2m})$, into a lower-dimensional space of observables. According to the prevalence version of Whitney’s embedding theorem [73], almost all sets of independent and simultaneous measurements $\text{y}(t)=(y_{1}(t),y_{2}(t),\ldots y_{p}(t))\in R^{p}$ form an embedding space for 2m-dimensional SSMs if p>4m. This is the case, for example, when displacements and velocities of at least 3m material points of a mechanical system are available. Practical experiments, however, generally only provide the displacement, velocity or acceleration of a single material point, denoted as s(t)∈R, recorded at Δt time intervals. To this end, we exploit Takens's delay embedding theorem [74], which, in its prevalence version [73], states that $\text{y}(t)=(s(t),s(t+\Delta t),s(t+2\Delta t),\ldots s(t+(p-1)\Delta t))\in R^{p}$ forms an embedding space with probability one if p>4m under generic non-degeneracy conditions on the sampling time Δt. Further spaces may also qualify in practice, e.g. featuring p≤4m or constructed from multiple measurements augmented by delays, but one needs to examine on a case-by-case basis whether these are embedding spaces or not.

We denote by $M_{0}$ the embedded SSM, for which we now need to construct a reduced-order model in the embedding space. We assume that the equilibrium is at y=0 and that $M_{0}$ does not fold over its tangent space at the origin $T_{\text{0}}M_{0}$, so that we can construct a data-driven graph-style parametrization for $M_{0}$ over $T_{\text{0}}M_{0}$. We let ${\text{V}}_{1}\in R^{p\times 2m}$ be the matrix whose orthonormal columns span $T_{\text{0}}M_{0}$ and we define the SSM parametrization, $\text{v}:R^{2m}\to R^{p}$, as

2.4 $$\text{y}=\text{v}({\text{V}}_{1}^{⊤}\text{y})={\text{V}}_{1}{\text{V}}_{1}^{⊤}\text{y}+{\text{v}}_{\mathrm{nl}}({\text{V}}_{1}^{⊤}\text{y}),\;{\text{V}}_{1}^{⊤}{\text{V}}_{1}=\text{I},\;{\text{V}}_{1}^{⊤}{\text{v}}_{\mathrm{nl}}({\text{V}}_{1}^{⊤}\text{y})=\text{0},$$

where we assume that ${\text{v}}_{\mathrm{nl}}:R^{2k}\to R^{p}$ is a multivariate polynomial from order 2 to M. The matrix ${\text{V}}_{1}$, as well as the coefficients of the polynomial ${\text{v}}_{\mathrm{nl}}$, can be found via constrained maximum-likelihood estimation of (2.4), as discussed in [63].

Once trajectories in the projection coordinates ${\text{V}}_{1}^{⊤}\text{y}\in R^{2m}$ are known, we can identify the SSM-reduced dynamics. Here, the idea is to find the extended normal form of the vector field governing the dynamics in the projection coordinate (or reduced) domain [63], motivated by classic studies of bifurcations [75,76]. Specifically, we need to find an invertible change of coordinates ${\text{V}}_{1}^{⊤}\text{y}=\text{h}(\text{z})$ (and its inverse) that brings the SSM-reduced dynamics to its simplest possible complex polynomial form $\dot{\text{z}}=\text{n}(\text{z})$ with $\text{z}\in C^{2m}$. The linear part of n is the diagonal matrix of the eigenvalues related to the SSM, with $\text{z}=(z_{1},{\bar{z}}_{1},z_{2},{\bar{z}}_{2},\ldots z_{m},{\bar{z}}_{m})$ denoting complex modal coordinates for the linearized system. The maps h, ${\text{h}}^{-1}$ and n are multivariate polynomials with their coefficients determined from an extended normal form approach used in classic unfoldings of bifurcations [75,76]. In this approach, the classic Poincaré [77] normal form construct is relaxed in that not only resonant but also near-resonant terms are kept in the normal form (see [63,78] for more details). This normalization renders n a sparse vector field extracting the fundamental physics, as we discuss in the next section. We determine resonant coefficients from an initial estimate of the linearized dynamics, and we identify from data the maps h, ${\text{h}}^{-1}$ and n by minimizing the conjugacy error, as explained in detail in [63]. For example, the structure of the cubic normal form for a two-dimensional SSM is

2.5 $$\begin{array}{rl} & \text{z}=(z,\bar{z}),\;\text{h}(\text{z})=(h_{1},{\bar{h}}_{1}),\;\text{n}(\text{z})=(n_{1},{\bar{n}}_{1}),\\ & h_{1}(\text{z})=z+h_{20}z^{2}+h_{11}z\bar{z}+h_{02}{\bar{z}}^{2}+h_{30}z^{3}+h_{12}z{\bar{z}}^{2}+h_{03}{\bar{z}}^{3},\;n_{1}(\text{z})=\lambda z+\gamma z^{2}\bar{z}, \end{array}$$

which resembles the classic Hopf normal form [79]. These normal form models are particularly simple to handle in polar coordinates $\left(\rho_{j},\theta_{j}\right)$, defined as $z_{j}=\rho_{j}\;e^{i\theta_{j}}$ for j=1,2,…,m.

### (b) Interpretability and extrapolation from spectral submanifold-reduced models

The most general normal form on a 2m-dimensional SSM is

2.6 $$\begin{array}{l} {\dot{\rho }}_{j}=-\alpha_{j}(\rho ,\theta )\rho_{j},\\ {\dot{\theta }}_{j}=\omega_{j}(\rho ,\theta ), \end{array}\;j=1,2,\ldots ,m,\;\rho =(\rho_{1},\rho_{2},\ldots \rho_{m}),\;\theta =(\theta_{1},\theta_{2},\ldots \theta_{m}).$$

Some explicit examples are presented in the examples of §3, including cubic polar normal forms of two-dimensional and four-dimensional SSMs, the latter appearing both for non-resonant eigenvalues and for a 1:2 resonance. If the linearized frequencies are non-resonant, then $\alpha_{j}$ and $\omega_{j}$ only depend on the amplitudes ρ. The normal form (2.6) then decouples the amplitude dynamics from the phase dynamics. This enables us to distinguish different modal contributions, perform a slow–fast decomposition, detect modal interactions and analyse the uncoupled oscillator limit. The zero-amplitude limit of the functions $\alpha_{j}$ and $\omega_{j}$ converges to the linearized damping and frequency of mode j, i.e.

2.7 $$\lim_{||\rho ||\to 0}[-\alpha_{j}(\rho ,\theta )+i\omega_{j}(\rho ,\theta )]=\lambda_{j}.$$

Hence, $\alpha_{j}$ and $\omega_{j}$ are the nonlinear continuations of these linear quantities, characterizing how dissipation and frequency change with respect to the amplitudes (and phases for internally resonant systems). For a two-dimensional SSM, the parametrized curves α(ρ) and ω(ρ) are the backbones of transient oscillations [1,8,54,57], representing the instantaneous damping and frequency as nonlinear functions of the normal form amplitude ρ. Normal form amplitudes do not, however, have any direct physical meaning. For physical insights, we need to express any amplitude of interest via the SSM parametrization v and the normal form transformation h. For instance, for two-dimensional SSMs and for a scalar quantity $g:R^{p}\to R$ defined on the observable space $R^{p}$, the amplitude of the oscillations can be defined as [54,56]

2.8 $$\mathrm{amp}(\rho )=\max_{\theta \in [0,2\pi )}|g(\text{v}(\text{h}(\text{z})))|,\;\text{z}=(\rho \;e^{i\theta },\rho \;e^{-i\theta }).$$

Then, backbone curves can be expressed as parametric curves {α(ρ),amp(ρ)} and {ω(ρ),amp(ρ)}.

SSMs are robust features of the dynamics, because they survive under small autonomous perturbations and even under some non-autonomous perturbations of the vector field (2.2) [52]. The most important class of these perturbations in our context is that of small external time-periodic forcing appearing on the right-hand side of equation (2.1). In that case, the autonomous SSM will serve as the leading order approximation for a non-autonomous, time-periodic SSM that carries reduced, time-periodic dynamics [52,57,58,60]. With the addition of such forcing, the normal form (2.6) becomes [63]

2.9 $${\dot{\rho }}_{j}=-\alpha_{j}(\rho ,\theta )\rho_{j}-f_{j}\sin(\Omega t-\theta_{j}),\;{\dot{\theta }}_{j}=\omega_{j}(\rho ,\theta )+\frac{f_{j}}{\rho_{j}}\cos(\Omega t-\theta_{j}),$$

where Ω is the forcing frequency and $f_{j}$ the forcing amplitudes for each mode. Generally, numerical continuation is necessary for studying periodic responses and eventual bifurcations of (2.9) depending on forcing frequencies and amplitudes. In the simplest case of m=1, however, we can introduce the phase shift ψ=θ−Ωt to obtain from (2.9) the forced normal form

2.10 $$\dot{\rho }=-\alpha (\rho )\rho +f\sin(\psi ),\;\dot{\psi }=\omega (\rho )-\Omega +\frac{f}{\rho }\cos(\psi ),$$

which yields closed-form predictions for amplitudes and phases of the forced periodic solutions

2.11 $$\Omega =\omega (\rho )\pm \sqrt{\frac{f^{2}}{\rho^{2}}-\alpha^{2}(\rho )},\;\psi =\tan^{-1}\left(\frac{\alpha (\rho )\rho }{\Omega -\omega (\rho )}\right),$$

known as frequency response curves (FRCs), parametrized by the amplitude ρ. Predictions of these curves from unforced data, however, have generally been unavailable. Physical amplitudes can be derived from the predictions of (2.11) using equation (2.8) and the stability of the predicted forced response can be derived from the Jacobian of the vector field (2.10) [57]. We find from equation (2.11) that the forced backbone curve (the location of maximal amplitude responses of FRCs under varying f) coincides with that of decaying oscillations, given by ω(ρ). Specifically, maximal amplitude responses occur at amplitudes $\rho_{\max}$ satisfying $f=\alpha (\rho_{\max})\rho_{\max}$, $\Omega =\omega (\rho_{\max})$ and phase-lag quadrature, i.e. θ=Ωt−π/2. These maximal amplitude responses can be used to calibrate the normal form forcing amplitude f with experimentally exerted forcing levels.

Equations (2.9) and (2.10) have O(fρ) accuracy [57,63], but higher-order approximations can improve this accuracy further [59]. We expect, for example, that forced backbone curves depart from those of decaying oscillations at large motion and/or large forcing amplitude values [80,81]. From a data-driven perspective, once the autonomous core of equations (2.9) and (2.10) is identified, we only need to calibrate the forcing amplitudes for predicting FRCs. In matching experimental results, one calibration point is sufficient if the forcing amplitude is kept constant during experimental frequency sweeps. The change of coordinates of the SSM normal form with forcing is also modulated by a small time-periodic component [57,59,63], i.e.${\text{V}}_{1}^{⊤}\text{y}=\text{h}(\text{z})+{\text{h}}_{f}(t,\Omega )$. For the two-dimensional SSMs example of (2.5), we then recall that we find ${\text{h}}_{f}(t,\Omega )=(if\;e^{-i\Omega t}{\left(\lambda +i\Omega \right)}^{-1},-if\;e^{i\Omega t}{\left(\bar{\lambda }-i\Omega \right)}^{-1})$.

## 3. Examples

We now discuss some examples that illustrate the power of the SSM-based, data-driven model reduction method we have discussed. Our first example is a chain of lumped oscillators, while the other two involve data from laboratory experiments. Additional details and further examples can also be found in [63] and in the Matlab^®^ live-scripts of the SSMLearn repository.

To express trajectory reconstruction errors, we use the normalized mean trajectory error NMTE, which, for a dataset of P instances of observable points ${\text{y}}_{j}\in R^{p}$ and their reconstruction $\hat{\text{y}}$, is defined as

3.1 $$\mathrm{NMTE}=\frac{1}{P||\underline{\text{y}}||}\sum_{j=1}^{P}||{\text{y}}_{j}-{\hat{\text{y}}}_{j}||.$$

Here, $\underline{\text{y}}$ is a relevant normalization vector, which is usually taken to be the data point ${\text{y}}_{j}$ with the maximum norm in the dataset. To validate the reduced dynamics on a test trajectory, we integrate the reduced-order model from the same initial condition and compare the results. Cross-validation is generally efficient in identifying the optimal polynomial order in SSMLearn after splitting the available data into training and testing trajectories.

### (a) Identification of spectral submanifolds in a chain of oscillators

**Figure 2.**
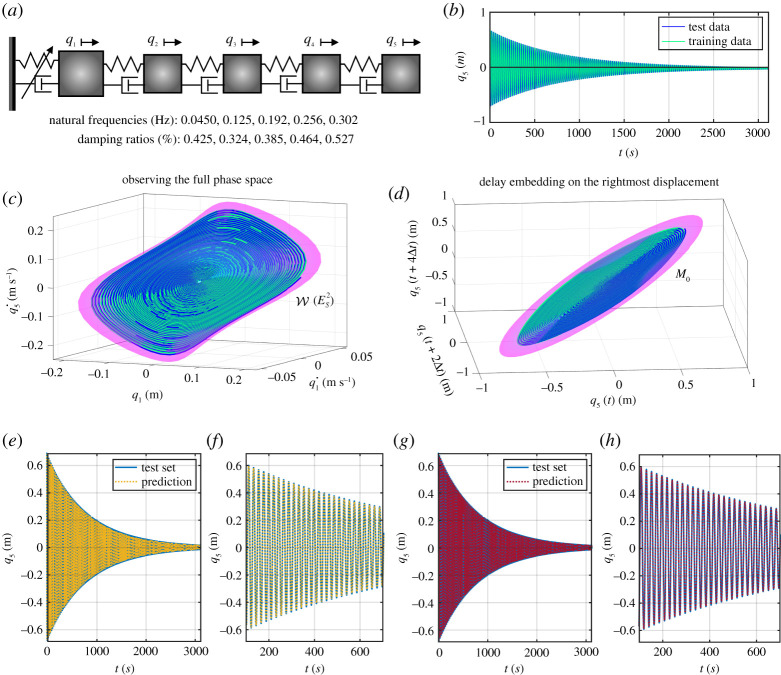
(*a*) Sketch of the oscillator chain considered in §3a. (*b*) Two trajectories decaying on its slow two-dimensional SSM. (*c*,*d*) The SSM and the trajectories in the phase space and in the delay observable space, respectively. (*e*–*h*) The performance of the normalized SSM-reduced models in reconstructing the test trajectory. (Online version in colour.)

We consider the chain of oscillators sketched in figure 2*a*, where we set the first mass as 1.5 kg and the others as 1 kg. We also assume all spring-dampers to be linear with unitary stiffness, except for the leftmost one that exerts a nonlinear force $f_{nl,1}=0.33{\dot{q}}_{1}^{2}+3q_{1}^{3}+0.7q_{1}^{2}\dot{q}+0.5{\dot{q}}_{1}^{3}$ on the first mass. The linear damping matrix for the system is proportional to the mass and stiffness matrices with constants 0.002 and 0.005, with the resulting eigenvalues at the trivial equilibrium reported in figure 2*a*.

We start with the study of the slow two-dimensional SSM $W(E_{S}^{2})$ of the oscillator chain. We compute this SSM via SSMTool [61], from which we initialize the two decaying trajectories shown in figure 2*b*; one of these trajectories is used for testing the constructed model. We identify reduced-order models from two different observables. The first observable set is the set of all phase space variables, while the second is a set of delayed samples of the (scalar) displacement of the rightmost mass $q_{5}$. We select the delay embedding of minimal dimension (five) required by the Takens theorem. The embedding coordinates are, therefore, $\text{y}(t)=(q_{5}(t),q_{5}(t+\Delta t),q_{5}(t+2\Delta t),q_{5}(t+3\Delta t),q_{5}(t+4\Delta t))$, where the sampling time Δt is 0.445 s. A cubic-order parametrization for the phase space embedding and a parametrization for the delay embedding show good accuracy. The SSM $W(E_{S}^{2})$ and its embedding in the delay space $M_{0}$ are shown in figure 2*b*,*c*. The flat appearance of the manifold in 2*d* in the delay space is a general phenomenon, as shown mathematically in [63]. The cubic polar normal form on the phase-space-embedded SSM is found by SSMLearn to be

3.2 $$\dot{\rho }=-0.001201\rho -0.0007300\rho^{3}=-\alpha (\rho )\rho ,\;\dot{\theta }=+0.2827+0.02546\rho^{2}=\omega (\rho ).$$

**Figure 3.**
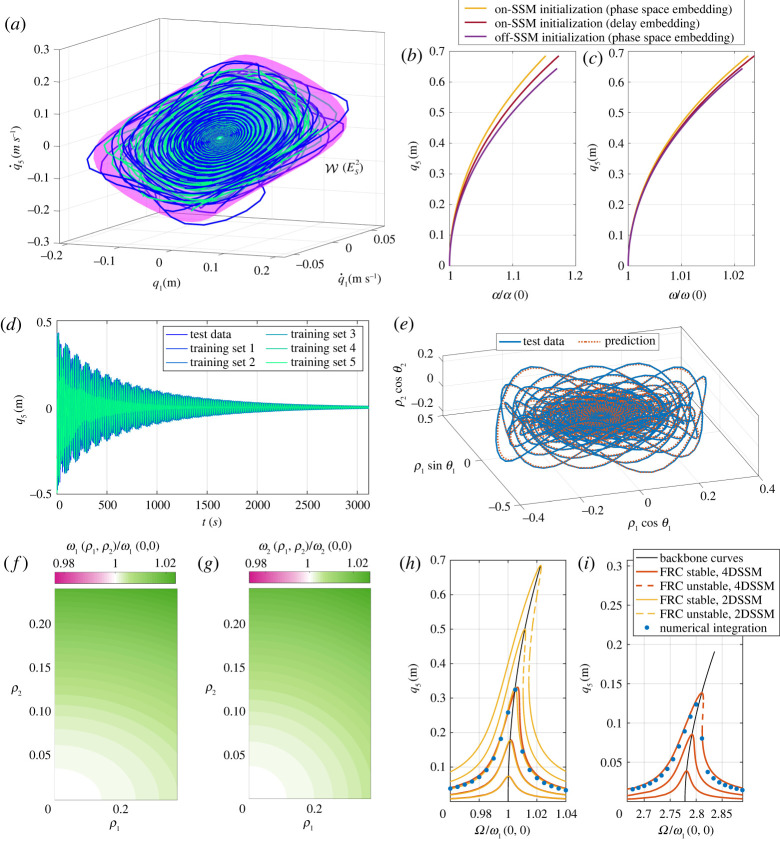
(*a*) Two trajectories converging to the slowest two-dimensional SSM of the oscillator chain. (*b*,*c*) Instantaneous damping and frequency curves constructed from a phase space embedding with perfect SSM initialization, from a delay embedding with perfect SSM initialization and from a phase space embedding with imperfect SSM initialization. (*d*) Decaying trajectories from the slow four-dimensional SSM $W(E_{S}^{4})$ of the oscillator chain. (*e*) Test trajectory and its model-based prediction in the normal form domain. (*f*,*g*) Instantaneous frequencies of the slow (mode 1) and fast (2) modes of $W(E_{S}^{4})$. (*h*,*i*) Frequency response curve (FRC) and backbone curves predictions from the reduced-order models (3.2) and (3.3) along with forced steady states (dots) obtained via numerical integration of the full system. (Online version in colour.)

A similar model is identified for the delay embedding. Both reduced-order models capture well the dynamics of the testing trajectories, as seen in figure 2*e*–*h*, with less than 2% NMTE error. The instantaneous damping α(ρ) and frequency ω(ρ) are shown in 3*b*,*c*, displaying only a minimal disagreement. We note that this identification is robust against perturbations of the initial condition. Indeed, if we initialize trajectories slightly off the SSM as shown in figure 3*a*, then SSMLearn still finds a good approximation for the reduced dynamics, as demonstrated by the curves in figure 3*b*,*c*. If these perturbations are not small enough for the dynamics to be described by two-dimensional SSMs, then we need to increase the SSM dimension. For instance, we computed trajectories, shown in figure 3*d*, decaying along the slow four-dimensional SSM, $W(E_{S}^{4})$. Five of these trajectories are used for training and one is left for testing our reduced-order model. The cubic normalized, SSM-reduced dynamics identified by SSMLearn has a 2.65% NMTE error and is of the form

3.3 $$\begin{array}{rl} & {\dot{\rho }}_{1}=-0.001200\rho_{1}-0.0005548\rho_{1}^{3}-0.01010\rho_{1}\rho_{2}^{2}=-\alpha_{1}(\rho_{1},\rho_{2})\rho_{1},\\ & {\dot{\rho }}_{2}=-0.002541\rho_{2}+0.003728\rho_{1}\rho_{2}^{2}-0.05627\rho_{2}^{3}=-\alpha_{2}(\rho_{1},\rho_{2})\rho_{2},\\ & {\dot{\theta }}_{1}=+0.2825+0.01316\rho_{1}^{2}+0.1085\rho_{2}^{2}=\omega_{1}(\rho_{1},\rho_{2}),\\ & {\dot{\theta }}_{2}=+0.7850+0.02340\rho_{1}^{2}+0.2760\rho_{2}^{2}=\omega_{2}(\rho_{1},\rho_{2}). \end{array}\}$$

The sparsity of the vector field (3.3) is guaranteed by the concept of extended normal forms [63], computed here for two weakly damped non-resonant, oscillatory modes. Eventual differences between the dynamics of the slowest mode in (3.2) with respect to those in (3.3) are due to different amplitude scalings. Prediction of a test trajectory based on the model (3.3) is shown in figure 3*e*. The instantaneous frequencies for the two modes are shown in figure 3*f*,*g*. These are surfaces since both frequencies depend (either weakly or strongly) on both modal amplitudes.

For additional validation, we show FRCs of the models (3.2)–(3.3) for different forcing amplitudes in figure 3*h*,*i* around the first two eigenfrequencies. While on the two-dimensional SSM $W(E_{S}^{2}),$ we have the closed-form solution (2.11), FRCs on the four-dimensional SSM $W(E_{S}^{4})$ are computed using the periodic orbit toolbox of the numerical continuation core coco [82]. These plots in figure 3*h*,*i* are completed with backbones curves and forced responses obtained via numerical integration of the full model. The forcing only acts in the direction of the first two modes, with amplitudes 0.38 and 1.75 mN. Our data-driven predictions, which are based only on unforced data and a simple calibration procedure for the normal form forcing amplitudes, are in close agreement with the responses from the full system.

### (b) Resonance decay in the Brake–Reuss beam

**Figure 4.**
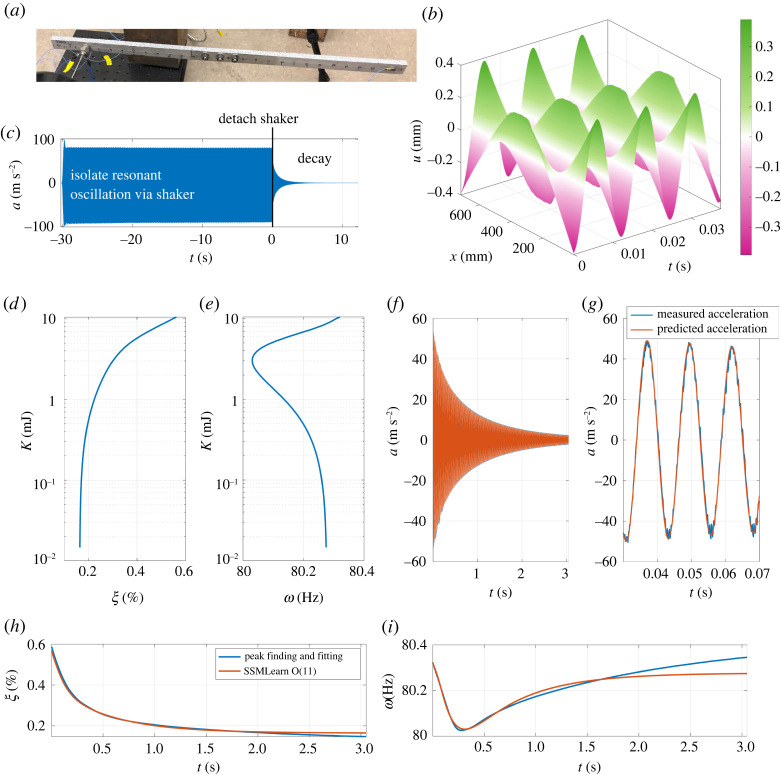
(*a*) Testing set-up for the Brake–Reuss beam. (*b*,*c*) The measured displacement and acceleration data. (*d*–*i*) Results from the reduced-order model trained on displacement data. The backbone curves in (*d*,*e*) show the instantaneous characteristics of the beam with respect to its kinetic energy, while (*f*,*g*) validate the predictions of acceleration. Plots (*h*,*i*) compare the instantaneous properties of the data-driven model with respect to those measured with the peak fitting and finding method [66] on the acceleration signal. (Online version in colour.)

The Brake–Reuss beam (BRB) is a benchmark system in the study of jointed structures [7,8,83]. In our study, it consists of two 304 stainless steel beams assembled with a lap joint, as shown in figure 4*a*. While full models for these structures may not be smooth, we find that trajectory data can be fitted well to smooth models, thereby justifying an SSM-based approach. The data considered here arise from a single resonance decay test, available from [8], targeting the slowest structural mode. One observable is the measurement from an accelerometer mounted, as shown in figure 4*a*, on the shaker with time history illustrated in figure 4*c*. Another observable is the displacement field of the bottom side of the beam, measured using DIC. The latter dataset, consisting of 206 points over 72 cm of beam length, has a limited time range due to limitations in camera memory. The initial evolution of the measured displacement field is depicted in figure 4*b*.

Our goal in this example is to construct a nonlinear reduced-order model using displacement data and validate it on the acceleration measurement. We truncate the time signals after shaker release to eliminate the influence of disturbances from non-perfect detachment. Nevertheless, high-frequency contributions decay rapidly and the transient settles along the slowest SSM. To diversify the data, we augment the displacement with four delayed measurements, so that the observable phase space has dimension 1030. The SSM is approximately a plane in this space, but the reduced dynamics is highly nonlinear. For adequate accuracy, the normal form indeed needs terms up to O(11) to capture the dynamics

3.4 $$\begin{array}{rl} & \dot{\rho }=-0.8255\rho -16.05\rho^{3}+166.3\rho^{5}-1421\rho^{7}+5314\rho^{9}-7138\rho^{11}=-\alpha (\rho )\rho ,\\ & \dot{\theta }=+504.4-46.16\rho^{2}+350.3\rho^{4}+412.9\rho^{6}-8468\rho^{8}+16975\rho^{10}=\omega (\rho ). \end{array}\}$$

The model can be used to approximate the beam kinetic energy as

3.5 $$K=\frac{1}{2}\frac{m_{\mathrm{BRB}}}{N_{\mathrm{DIC}}}\sum_{j=1}^{N_{\mathrm{DIC}}}{\dot{u}}_{j}^{2}(t),$$

where $N_{\mathrm{DIC}}=206$ is the number of DIC measurement locations and $m_{\mathrm{BRB}}=1.796\;\text{kg}$ is the beam mass. As discussed in [8], the kinetic energy amplitude is a good proxy for the instantaneous decay properties, i.e. the instantaneous damping ratio and frequency, shown in figure 4*d*,*e*, respectively. The instantaneous damping ratio is defined from the normal form dynamics (3.4) as ξ(ρ)=α(ρ)/ω(ρ), expressed in percentage. The damping exhibits a strong variation from its linear limit, while the frequency here shows a peculiar softening–hardening trend. We note that lower-order models for the dynamics fail to capture the softening–hardening trend shown in figure 4*e*,*i*. Indeed, one needs at least quintic order for modelling such a trend, and higher orders tend to increase the accuracy. At the same time, excessively higher-order approximations generally lead to overfitting the data.

We validate our displacement-based SSM-reduced model on the data from the accelerometer located at 77 mm from the left end of the beam, as shown in figure 4*a*. This signal is reconstructed from the model by interpolating in the grid to obtain the accelerometer location and differentiating in time. These predictions show good accuracy, as reported in figure 4*f*,*g*. A further validation in figure 4*h*,*i* compares the instantaneous decay properties of the data-driven model (3.4) to those extracted using the peak finding and fitting [8,66] signal processing technique. There is close agreement among these curves, especially in the strongly nonlinear oscillation regime.

### (c) Impacts on an internally resonant tester structure

**Figure 5.**
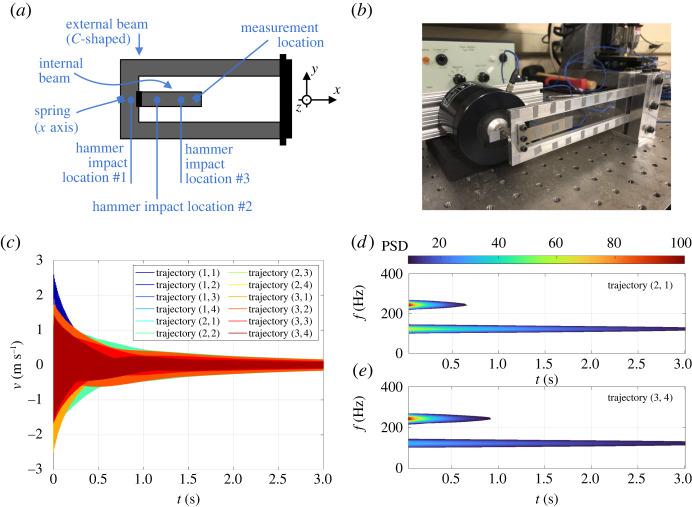
(*a*,*b*) Schematics and laboratory photo for the resonant tester. (*c*) Velocity time series of the inner beam tip. (*d*,*e*) Power spectral density computed via short-time Fourier transform (spectrogram) for two decaying responses of hammer impact tests. (Online version in colour.)

Our final example is the resonant tester shown in figure 5*a*,*b*. It consists of two beam-like parts made of aluminium 6061-T6, where the external beam is C-shaped and clamped to the ground on one side, while the internal beam is jointed to the external one via three bolts: two side bolts are torqued to 1.36 Nm for structural integrity whereas the middle bolt is torqued to 0.45 Nm for enhanced frictional slip and associated nonlinearities. Additionally, a linear spring (Model #1NCH2, Grainger, Inc.) connects the tip of the external beam to a fixed rigid frame in the direction of the *z*-axis. The system possesses an internal 1:2 resonance between its slowest transverse bending modes, whose frequencies indeed clock at 122.4 Hz and 243.4 Hz. We consider transverse vibrations in the out of plane direction—the *z*-axis in figure 5*a*. The available observable is the velocity of the inner beam tip, measured via laser scanner vibrometry (PSV400, Polytec Inc.). Transient vibrations are recorded for 3 s at a sampling rate of 5120 Hz.

A modally tuned impulse hammer (PCB 086C01, PCB Piezotronics, Inc.) is used to excite transverse vibrations from three different impact locations in figure 5*c*, so that our dataset features 12 trajectories (four per impact location), shown in figure 5*c*. We label these trajectories as (j,l) where j refers to the location and l to the test number. Time-frequency analyses of the velocity signals, two of which are reported in figure 5*d*,*e*, show that only the two slowest frequencies are present in the signal, so that the time responses can be well approximated by the slowest four-dimensional SSM of the system. The impact locations, the hammer tip and the forcing amounts were selected to achieve a sufficient trajectory diversity in the dataset without exciting further structural modes. For constructing an SSM-reduced model, we truncate the velocity signals after the hammer impact, use 10 trajectories for training and leave two trajectories for testing.

The minimal embedding dimensions (nine for a four-dimensional manifold) fail to produce accurate reduced-order models (the NMTE error amounts to more than 8%). We therefore augment the delay embedding space so that each embedding vector captures approximately two cycles of the slowest oscillation. This procedure yields a 94-dimensional delay embedding space. The result of our identification remains robust if we consider more embedding dimensions. A linear approximation to the embedded SSM has a good accuracy and our automated normal form algorithm, after estimating linearized eigenvalues, identifies a resonance among them. Defining $\psi =\theta_{2}-\theta_{1}$, we obtain from SSMLearn the cubic SSM-reduced polar normal form

3.6 ρ˙1=−0.4228ρ1−19.94ρ13+3.514ρ1ρ22+Re((0.08706−0.2427i)ρ2ρ1 eiψ) =−α1(ρ1,ρ2,ψ)ρ1,ρ˙2=−3.155ρ2−18.91ρ12ρ2−15.08ρ23+Re((1.726−0.3342i)ρ12 e−iψ) =−α2(ρ1,ρ2,ψ)ρ2,ρ1θ˙1=+769.0ρ1−59.56ρ13−0.5460ρ22ρ1+Im((0.08706−0.2427i)ρ2ρ1 eiψ) =ω1(ρ1,ρ2,ψ)ρ1,ρ2θ˙2=+1529ρ2−31.26ρ12ρ2−28.65ρ23+Im((1.726−0.3342i)ρ12 e−iψ) =ω2(ρ1,ρ2,ψ)ρ2.}

**Figure 6.**
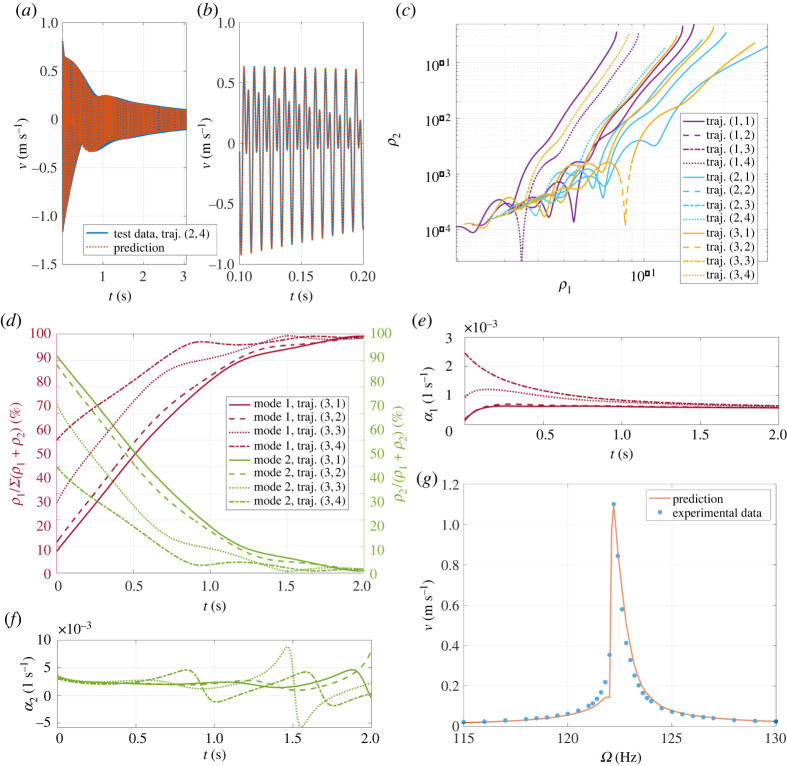
(*a*,*b*) Data-driven reduced-order model performances in reconstructing a testing trajectory. (*c*) Normal form amplitudes decays for the slow $\rho_{1}$ and fast $\rho_{2}$ modes, for all available trajectories in the dataset. (*d*) Energy repartition in the resonant tester following a hammer impact on the third location. (*e*,*f*) The trend of instantaneous (or effective) damping of the normal form dynamics on the first 2 s of decays related to the third impact location. (*g*) Forced frequency response from experimental measurements and from analytical predictions based on the SSM-reduced model constructed by SSMLearn. (Online version in colour.)

This data-driven model reconstructs both test trajectories with an average 1.2% NMTE error, cf. Figure 6*a*,*b*. The decay of the slow mode amplitude $\rho_{1}$ and that of the fast one $\rho_{2}$ are shown in figure 6*c*. Due to modal interactions, these decays are not monotone. From the plot, we note a great diversity of decays depending on the impact location, and location three (the closest to the inner beam tip) is characterized by the highest amplitudes variability.

Figure 6*d* shows energy repartition among the modes for the third impact location. This repartition is defined as the instantaneous ratio between the amplitude of a mode and the amplitude sum. Clearly, the slow mode tends to accumulate energy over time, while the fast mode dissipates it quickly. These trends are not monotonic, showing simultaneous and opposite changes in growth/decay rates, which implies that the faster mode is absorbing energy from the slower one. This behaviour can also be inferred by the instantaneous properties illustrated in figure 6*e*,*f*. The uncoupled limit of the oscillators suggests that the modes admit frequency softening and damping intensification when the oscillation amplitude increase. This is consistent with typical observations of jointed structures [83]. In particular, the fast mode is coupled to the slow one and its damping undergoes consistent variation, becoming also negative for some times [84]. Note that nonlinearity and coupling can be reduced at higher bolt torques, which, in the beam assembly used here, corresponds to tightening of the middle bolt. Coupling revealed by SSMLearn suggests that nearly decoupled modal oscillator models employed elsewhere [85,86] are only valid for high bolt torques and small frictional slip, i.e. weak contact nonlinearities.

In addition to measuring decaying vibrations, we also perform some forced testing near the linearized frequency of the slow mode. We trigger forced responses in near-resonance with the slow (first bending) mode by using the Brüel & Kjær 4810 shaker shown in figure 5*b*, mounted on one end of the linear spring, and acquire velocity response from the tip of the inner beam by laser vibrometry. We also monitor the amplitude of shaker tip velocities and keep them constant while sweeping the frequencies around the first bending mode. In that sense, the response we obtain can be seen as transmissibility rather than a classic FRC, with forcing amplitudes kept constant throughout frequency sweeps. Starting from forced velocity time histories, we estimate the normal form forcing to be added to the vector field (3.6) as in (2.9). The resulting predictions are in very good agreement with experimental measurements in this weakly nonlinear regime, as shown in figure 6*g*. Deeper analyses on forced responses are currently under investigation. Thanks to feedback loops used to track forcing, forced response curves can be extracted with improved accuracy, especially at nonlinearizable amplitudes.

## 4. Conclusion

We have reviewed a general methodology for constructing sparse reduced-order models for potentially high-dimensional, nonlinear mechanical systems from data. Our approach constructs normal forms on attracting SSMs, which are the smoothest nonlinear continuation of spectral subspaces of the linearized dynamics. Implemented in the publicly available Matlab^®^ code SSMLearn, our algorithm takes generic observable data as input to identify robust and predictive nonlinear models that also capture for nonlinearizable dynamics. SSM theory offers a systematic basis for model reduction and allows a simplification of the reduced dynamics via normal forms, which are particularly insightful for mechanical systems. Indeed, SSM-reduced models can handle multi-modal interactions, identify amplitude-dependent damping and frequency, and predict the forced structural response.

We have illustrated SSM-reduced modelling in numerical and experimental case studies, featuring different types of observables, nonlinearities and SSM dimensions. Specifically, we have discussed different dynamical regimes and the relevance of slow SSMs in a chain of oscillators, derived a reduced-order model from digital image correlation measurements of the BRB, and unfolded the internally resonant dynamics of a tester structure, also predicting forced responses. These examples were analysed using the open-source Matlab^®^ package SSMLearn that performs data-driven, SSM-based model reduction starting from vibrations data. This algorithm only requires a minimal number of input parameters: the SSM dimension, the polynomial order for SSM parametrization and the polynomial order of the reduced dynamics. The SSM dimension is either known *a priori* from targeted experiments (e.g. resonance decay) or can be estimated via time-frequency signal processing analysis of the input data. This makes our method a parametric complement to non-parametric identification tools. Polynomial orders can be adjusted to improve accuracy, noting that excessive orders may lead to overfitting. With the help of the numerical continuation core coco [82] included in SSMLearn, users can compute forced response curves or design nonlinear control strategies from the identified nonlinear models.

Further examples, both numerical and experimental, with detailed code are available in the SSMLearn repository, which is also suitable for high-dimensional fluid flows and fluid–structure interaction problems [63]. Current limitations of the present approach include weaker performance for large forcing amplitudes. These appear, for example, in the BRB experiments of [7,8], which we expect to capture only with a more refined forced-reduced dynamics and improved calibration procedures. The same requirement holds for more complicated forcing types (e.g. quasi-periodic or random), which are relevant in structural dynamics. Moreover, polynomial models, which are always a good approximation for near-equilibrium dynamics, may be limited in their ability to capture multi-scale dynamics arising from phenomena such as friction and wear. We are addressing these challenges in ongoing work.

## Data Availability

All data and code discussed in the results presented here are publicly available in the SSMLearn repository at github.com/haller-group/SSMLearn.
